# Supplementary material for: Endophytic Strain Bacillus subtilis 26D Increases Levels of Phytohormones and Repairs Growth of Potato Plants after Colorado Potato Beetle Damage
Source: Plants (Basel). 2021 May 5;10(5):923. doi: 10.3390/plants10050923 (PMC8148200; doi:10.3390/plants10050923)
Supplement: Supplementary file 1 [file plants-10-00923-s001.zip › plants-1179054-SI.pdf]

Table S1. Impact of CPB damage and *Bacillus subtilis* 26D treatment on the length of shoots and the rate of growth addition of shoots of potato plants. Data were analyzed using two-way ANOVA with CPB damage and *B. subtilis* 26D treatment as main factors. Degrees of freedom (df), sums of squares (SS), mean squares (MS) F values, P values are presented.

	Length of shoots						
	Source of Variation	***SS***	***df***	***MS***	***F***	***P-value***	***F crit***
0 day	CPB	0,0188	1	0,0188	0,0288	0,8657	3,9229
	B.s. 26D	1,8008	1	1,8008	2,7613	0,0993	3,9229
	CPB+*B.s.* 26D	0,6021	1	0,6021	0,9233	0,3386	3,9229
	Within	75,6463	116	0,6521			
	Total	78,06792	119				
1 day	CPB	5,7203	1	5,7203	5,7754	0,0178	3,9229
	B.s. 26D	1,4083	1	1,4083	1,4219	0,2355	3,9229
	CPB+*B.s.* 26D	10,6803	1	10,6803	10,7831	0,0014	3,9229
	Within	114,8947	116	0,9905			
	Total	132,7037	119				
3 day	CPB	9,5768	1	9,5768	7,7745	0,0519	3,9229
	B.s. 26D	4,0701	1	4,0701	3,3041	0,0717	3,9229
	CPB+*B.s.* 26D	71,3021	1	71,3021	57,8836	8,02E-12	3,9229
	Within	142,8910	116	1,2318			
	Total	227,8399	119				
6 day	CPB	15,0238	1	15,0238	15,4685	0,0001	3,9229
	B.s. 26D	32,3856	1	32,3856	1,3444	6,5763	3,9229
	CPB+*B.s.* 26D	7,3310	1	7,3310	3,5480	0,07	3,9229
	Within	112,6647	116	0,9713			
	Total	167,405	119				
9 day	CPB	28,3241	1	28,3241	20,1366	1,71E-05	3,9229
	B.s. 26D	4,3701	1	4,3701	3,1068	0,0806	3,9229
	CPB+*B.s.* 26D	12,6101	1	12,6101	8,9649	0,0034	3,9229
	Within	163,1657	116	1,4066			
	Total	208,4699	119				
	Growth addition of shoots						
	Source of Variation	***SS***	***df***	***MS***	***F***	***P-value***	***F crit***
1 day	CPB	7,9413	1	7,9413	101,3352	0,00001	3,9229
	B.s. 26D	0,1710	1	0,1710	2,1821	0,1423	3,9229

	CPB+*B.s.* 26D	0,9135	1	0,9135	11,6568	0,0009	3,9229
	Within	9,0905	116	0,0784			
	Total	18,1164	119				
3 day	CPB	2,3297	1	2,3297	4,9268	0,0284	3,9229
	B.s. 26D	1,6055	1	1,6055	3,3952	0,0679	3,9229
	CPB+*B.s.* 26D	6,5707	1	6,5707	13,8958	0,0003	3,9229
	Within	54,8515	116	0,4729			
	Total	65,3573	119				
6 day	CPB	0,2050	1	0,2050	4,5761	0,04494	3,9229
	B.s. 26D	1,4170	1	1,4170	3,9819	0,0483	3,9229
	CPB+*B.s.* 26D	15,7253	1	15,7253	44,1895	0,00001	3,9229
	Within	41,2798	116	0,3559			
	Total	58,6271	119				
9 day	CPB	0,4177	1	0,4177	2,0732	0,1526	3,9229
	B.s. 26D	0,1129	1	0,1129	0,5601	0,4557	3,9229
	CPB+*B.s.* 26D	0,7809	1	0,7809	3,8755	0,0514	3,9229
	Within	23,3723	116	0,2015			
	Total	24,6837	119				

Table S2. Impact of CPB damage and *Bacillus subtilis* 26D treatment on fresh (A) and dry (B) weights of shoots and roots of potato plants on the 9th day post damage caused by CPB. Data were analyzed using two-way ANOVA with CPB damage and *B. subtilis* 26D treatment as main factors. Degrees of freedom (df), sums of squares (SS), mean squares (MS) F values, P values are presented.

	Fresh weight						
	Source of Variation	***SS***	***df***	***MS***	***F***	***P-value***	***F crit***
Shoots	CPB	0,0117	1	0,0117	16,0046	0,0001	3,9229
	B.s. 26D	0,0007	1	0,0007	0,9440	0,3333	3,9229
	CPB+*B.s.* 26D	0,0070	1	0,0070	9,5810	0,0025	3,9229
	Within	0,0847	116	0,0007			
	Total	0,1041	119				
Roots	CPB	0,5548	1	0,5548	17,4352	0,0001	3,9229
	B.s. 26D	0,1769	1	0,1769	5,5595	0,0201	3,9229
	CPB+*B.s.* 26D	0,1982	1	0,1982	6,2295	0,0140	3,9229
	Within	3,6909	116	0,0318			

	Total	4,6208	119				
	Dry weight						
	Source of Variation	***SS***	***df***	***MS***	***F***	***P-value***	***F crit***
Shoots	CPB	0,000040	1	0,000040	11,6354	0,0010	3,9229
	B.s. 26D	0,000003	1	0,000003	1,0143	0,3160	3,9229
	CPB+*B.s.* 26D	0,000048	1	0,000048	14,2204	0,0003	3,9229
	Within	0,000394	116	0,000003			
	Total	0,000485	119				
Roots	CPB	0,0046	1	0,0046	17,5426	0,0001	3,9229
	B.s. 26D	0,0019	1	0,0019	7,2517	0,0081	3,9229
	CPB+*B.s.* 26D	0,0019	1	0,0019	7,3415	0,0078	3,9229
	Within	0,0301	116	0,0003			
	Total	0,0385	119				

Table S3. Impact of CPB damage and *Bacillus subtilis* 26D treatment on phytohormones level in potato plants. Data were analyzed using two-way ANOVA with CPB damage and *B. subtilis* 26D treatment as main factors. Degrees of freedom (df), sums of squares (SS), mean squares (MS) F values, P values are presented.

	IAA						
	Source of Variation	***SS***	***df***	***MS***	***F***	***P-value***	***F crit***
1 day	CPB	340,5485	1	340,5485	11,0593	0,0034	4,3512
	B.s. 26D	23,2611	1	23,2611	0,7554	0,3951	4,3512
	CPB+*B.s.* 26D	576,2457	1	576,2457	18,7135	0,0003	4,3512
	Within	615,8592	20	30,7929			
	Total	1555,914	23				
2 day	CPB	473,5345	1	473,5345	8,5863	0,0083	4,3512
	B.s. 26D	75,6617	1	75,6617	1,3719	0,2552	4,3512
	CPB+*B.s.* 26D	469,2916	1	469,2916	8,5094	0,0085	4,3512
	Within	1102,994	20	55,1496			
	Total	2121,481	23				
3 day	CPB	653,3870	1	653,3870	20,6482	0,0002	4,3512
	B.s. 26D	8,3205	1	8,3205	0,2629	0,6137	4,3512
	CPB+*B.s.* 26D	479,3294	1	479,3294	15,1477	0,0009	4,3512
	Within	632,8751	20	31,6438			
	Total	1773,9120	23				

	ABA						
	Source of Variation	***SS***	***df***	***MS***	***F***	***P-value***	***F crit***
1 day	CPB	134,9539	1	134,9539	7,0433	0,0152	4,3512
	B.s. 26D	8,2828	1	8,2828	0,4323	0,5184	4,3512
	CPB+*B.s.* 26D	94,4379	1	94,4379	4,9288	0,0381	4,3512
	Within	383,2096	20	19,1605			
	Total	620,8843	23				
2 day	CPB	273,1363	1	273,1363	13,70555	0,00141	4,351244
	B.s. 26D	89,7924	1	89,7924	4,505642	0,04646	4,351244
	CPB+*B.s.* 26D	195,6074	1	195,6074	9,815271	0,005238	4,351244
	Within	398,5776	20	19,92888			
	Total	957,1137	23				
3 day	CPB	160,9644	1	160,9644	7,1766	0,0144	4,3512
	B.s. 26D	57,3328	1	57,33281	2,5562	0,1255	4,3512
	CPB+*B.s.* 26D	176,9009	1	176,9009	7,8872	0,0109	4,3512
	Within	448,5771	20	22,4289			
	Total	843,7752	23				
	Zeatin						
	Source of Variation	***SS***	***df***	***MS***	***F***	***P-value***	***F crit***
1 day	CPB	4,4235	1	4,4235	11,5636	0,0028	4,3512
	B.s. 26D	69,7389	1	69,7389	4,07781	0,0590	4,3512
	CPB+*B.s.* 26D	168,7756	1	168,7756	0,3031	0,5881	4,3512
	Within	291,9088	20	14,5954			
	Total	534,8468	23				
2 day	*CPB*	586,3321	1	586,3321	8,319744	0,009168	4,351244
	B.s. 26D	64,52952	1	64,52952	0,91564	0,350047	4,351244
	CPB+B.s. 26D	776,5493	1	776,5493	11,01883	0,00342	4,351244
	Within	1409,496	20	70,47478			
	Total	2836,906	23				
3 day	*CPB*	4219,952	1	4219,952	21,38168	0,000164	4,351244
	B.s. 26D	5099,518	1	5099,518	25,83826	5,68E-05	4,351244
	CPB+B.s. 26D	3536,128	1	3536,128	17,91688	0,000408	4,351244
	Within	3947,26	20	197,363			
	Total	16802,86	23				

	Zeatin-ribozid						
	Source of Variation	***SS***	***df***	***MS***	***F***	***P-value***	***F crit***
1 day	CPB	196,8381	1	196,8381	9,5902	0,0057	4,3512
	B.s. 26D	225,4229	1	225,4229	10,9829	0,0035	4,3512
	CPB+*B.s.* 26D	24,4268	1	24,4267	1,1901	0,2883	4,3512
	Within	410,4985	20	20,5249			
	Total	857,1862	23				
2 day	CPB	432,1788	1	432,1788	76,19944	2,95E-08	4,3512
	B.s. 26D	35,9567	1	35,95669	6,339689	0,020437	4,3512
	CPB+*B.s.* 26D	27,4529	1	27,45286	4,840341	0,039722	4,3512
	Within	113,4336	20	5,67168			
	Total	609,022	23				
3 day	CPB	760,9784	1	760,9784	0,0133	0,9094	4,3512
	B.s. 26D	30,1057	1	30,1057	4,4428	0,0479	4,3512
	CPB+*B.s.* 26D	0,09	1	0,09	112,2992	1,18E-09	4,3512
	Within	135,527	20	6,7764			
	Total	926,7011	23				
